# RETRACTION: Preliminary Evaluation of the Value of a Small‐Molecule Probe Targeting DNMT1 in Detecting the Methylation of PAX1 in Cervical Cancer

**DOI:** 10.1111/srt.70157

**Published:** 2025-01-07

B. Yang, Q. Cheng, X. Hong, X. Zhu, Z. Xia, W. Chen, and L. Xu, “Preliminary Evaluation of the Value of a Small‐Molecule Probe Targeting DNMT1 in Detecting the Methylation of PAX1 in Cervical Cancer,” *Skin Research and Technology* 30, no. 10 (2024): e70074, https://doi.org/10.1111/srt.70074.

The above article, published online on 27 September 2024 in Wiley Online Library (wileyonlinelibrary.com), has been retracted by agreement between *Skin Research and Technology*; and John Wiley & Sons Ltd. The article was submitted as part of a guest‐edited special issue. Following publication, it has come to the attention of the journal that the article was accepted solely on the basis of compromised editorial handling and peer review processes. Furthermore, the article is outside the journal's scope, presenting irrelevant citations and conclusions not supported by the data. Finally, another article with several authors in common was published in the same special issue, sharing concepts, methodology, and data.

Therefore, the decision to retract this article was taken.

